# Maturity-Related Variations in Morphology, Body Composition, and Somatotype Features among Young Male Football Players

**DOI:** 10.3390/children10040721

**Published:** 2023-04-13

**Authors:** Denis Čaušević, Babina Rani, Qais Gasibat, Nedim Čović, Cristina Ioana Alexe, Silviu Ioan Pavel, Lucian Ovidiu Burchel, Dan Iulian Alexe

**Affiliations:** 1 Faculty of Sport and Physical Education, University of Sarajevo, 71000 Sarajevo, Bosnia and Herzegovina; denis.causevic@fasto.unsa.ba (D.Č.); nedim.covic@fasto.unsa.ba (N.Č.); 2 Department of Physical Rehabilitation & Medicine (Physiotherapy), Post Graduate Institute of Medical Education and Research, Chandigarh 160012, India; says2babina@gmail.com; 3 Department of Sports Studies, Universiti Putra Malaysia UPM, Serdang 43400, Malaysia; gs57022@student.upm.edu.my; 4 Department of Physical Education and Sports Performance, Faculty of Movement, Sports and Health Sciences, “Vasile Alecsandri” University of Bacău, 600115 Bacău, Romania; alexe.cristina@ub.ro; 5 Department of Environmental Sciences, Physics, Physical Education and Sports Faculty of Sciences, Lucian Blaga University in Sibiu, 550024 Sibiu, Romania; 6 Department of Physical and Occupational Therapy, Faculty of Movement, Sports and Health Sciences, “Vasile Alecsandri” University of Bacău, 600115 Bacău, Romania; alexedaniulian@ub.ro

**Keywords:** team sports, youth athletes, soccer, biological age

## Abstract

The study aimed to investigate differences in anthropometry, body composition (BC), and somatotype in young football players of the same chronological age according to the maturity stage. Overall, 64 elite players (age: 14.28 ± 0.46 years) were evaluated for standing and sitting body height, girth measures, and BC using the bioelectric impedance scale and skinfold thickness. In total, two-thirds (73.44%, n = 47) of football players were classified as on-time maturers, 12.50% (n = 8) were early maturing, and 14.06% (n = 9) were late maturing. Standing and sitting height, leg length, fat-free mass, and muscle mass were significantly different (*p* < 0.001) across maturity groups. A significant decrease (*p* < 0.05) with maturity progression was seen for subscapular and suprailiac skinfolds along with a girth increase at all sites (*p* < 0.05). Early maturers were balanced ectomorphs, while on-time and late maturers featured mesomorph–ectomorph characteristics. The obtained results suggested that mature players have better BC presented as a lower fat percentage along with higher muscle mass, advantages in circumferences, and longitudinal dimensions of the body with highlighted mesomorph features. Maturity can have a substantial influence on body measures, thereby affecting sport-specific performance. Early maturers can use their anthropometric advantages and compensate for a lack of talent, consequently preventing even participation of physically undeveloped players in training. A better understanding of maturity, BC, and somatotypes can help in the selection of young talented players.

## 1. Introduction

Football is a sport that has a few key requirements in order to compete at the highest level possible. This is true regardless of the sort of competition, such as age, categories, and playing positions. [1]. There are several factors that must be taken into account in order to perform at the maximum level, including anthropometry and physical and physiological characteristics.

Previous studies [2,3,4] have documented that an individual’s body dimensions, diameters, skinfolds, and other anthropometric profile parameters are all considered crucial elements of an elite football player’s somatic build profile. In this regard, scientific research on these topics has advanced, but it still provides comprehensive information, particularly for young age categories. Young football players need to be distinguished from one another because any category can comprise players of different chronological and biological ages. [5]. As a result, maturity, body composition, anthropometry, and somatotype parameters are required to determine the player’s current state.

Anthropometric measures and body composition can vary among different sports based on the physical demands and requirements of the sport. This is one of the reasons why they play a significant role in determining an athlete’s physical abilities and performance [6]. Previous studies have shown that the anthropometric and body composition profile of football players has been associated with various match performances, especially body-fat percentage [7], fat-free mass [8], and weight and height [9].

Along with their anthropometric characteristics, a player’s morphology is a significant and trustworthy predictor of their success. Somatotype is a vital component of an athlete’s physical body structure [10] and has a significant impact on how well they execute on the field. Somatotypes are classified through relative fatness (endomorphy), musculoskeletal component (mesomorphy), and linearity (ectomorphy) [11]. In football, a player’s somatotype can significantly impact their ability to execute specific plays and compete effectively against opponents. Balanced mesomorphs are presented as superior types at performing football-specific activities with respect to their functional needs, power, speed, and agility. Defensive players and forwards tend to be mesomorph–ectomorphs with an advantage in head play due to the frequent jumps they must perform during tactical tasks [12]. In contrast, midfielders’ somatotype characteristics help them during dribbling, handling the ball, moving more efficiently, and allowing them to cover greater distances on the field [13]. Despite this, the ideal somatotype for football players varies based on the physical demands of their positions on the field [3,14]; football players tend to be healthy mesomorphs with high levels of muscularity and low levels of body fat [5].

Given that the chronological and biological ages of young football players may differ, calculating the biological age is critical [15], and it encourages the use of physiological and fitness indicators in addition to anthropometric measurements to determine the player’s functional state [4].

According to scientific research, maturity level is a key factor in recognizing and choosing talented young football players [16,17,18]. To choose highly skilled teenage football players, maturity status, a crucial indication of biological development, should be considered [19]. Moreover, a young football player’s maturity level may have an impact on their physical condition throughout the season and their career [20]. The time needed to achieve adulthood is then determined by biological maturity. During this time, many sexual, morphological, neural, hormonal, somatic, and skeletal changes may occur [4,21]. The age at which the greatest height rise occurs is known as the projected maturity offset, and it is marked by accelerated genetically-related body composition changes. Age at peak height velocity (PHV) is a term used to describe this component [22]. A greater variance in body height, ranging from 8.2 to 10.3 cm annually, was noted during the PHV stage [23]. According to research, adolescent male football players typically develop PHV between the ages of 14 and 15 [24,25]. During the PHV stage, a significant body mass increase was observed as well. Body mass presents one of the most influencing factors concerning football performance since it impairs improvements in activities, such as jumping, sprinting, and running.

For instance, Goto et al. [26] discovered that more mature players had a greater opportunity to participate in U9 and U10 competitions due to their body size advantage. It is possible that the more mature players had a physical advantage over their less mature peers, which allowed them to perform better and be selected for these competitions. However, further research would be needed to fully understand the reasons behind this phenomenon. Therefore, it also becomes imperative to investigate whether body maturity status affects somatotype features in a similar way among players at the PHV stage and to what extent the presence of early maturers exists. Therefore, the aim of the present study was to investigate the differences in anthropometric measures and body composition characteristics in relation to the biological maturation of elite young football players (U15).

## 2. Materials and Methods

### 2.1. Participants

Sixty-four elite male football players U15 (age: 14.28 ± 0.46 years) participated in the study. All had a minimum of 4 years of playing experience and were registered in a professional Bosnia and Herzegovina football team participating in the first division. In order to analyze the differences in biological maturation, they were classified into three groups: early maturers (n = 8); average (on time) maturers (n = 47), and late maturers (n = 9).

### 2.2. Experimental Design

This was a transversal observational and descriptive study. Exclusion criteria in this study were (1) players who failed to participate in at least 80% of training seasons in the last year; (2) players from divisions different from the first division; (3) players that had an injury in past three months; and (4) players with any active injuries. The study was approved by the Ethics Committee of the Faculty of Sports and Physical Education, University of Sarajevo (N0: 01-2603/22; 1 July 2022.) and was carried out in accordance with the Declaration of Helsinki. Upon explaining the procedures, parents signed written consent allowing the minor subjects to participate in the study, which guaranteed full ethical consideration and the possibility to withdraw at any time.

### 2.3. Procedures

Body composition with anthropometric measurements was performed by trained personnel according to standardized procedures. For this study, specific testing was designed, which included one-day testing planned in a specific order and at the same time of the day to avoid any influence of the diurnal variations. The on-field testing session was performed by an expert in the field of physical education. All the testing sessions were performed at the Institute of Sport, Faculty of Sport and Physical Education, University of Sarajevo, in the morning between 09:00 and 12:00. The testing was performed in a single session per player. Standing and sitting body height were measured to one decimal place (0.1 cm) using a digital stadiometer (InBody BSM 370; Biospace Co., Ltd., Seoul, Republic of Korea), and leg length was derived by the subtraction of sitting height from standing height. Body weight, fat-free mass (FFM), muscle mass (MM), body mass index (BMI) and body fat percentage (BF%) were estimated to the nearest 0.1 kg (underwear, barefoot) using a direct segmental high-frequency bioelectrical impedance scale (InBody 720; Biospace Co., Ltd., Seoul, Republic of Korea). Skinfold thickness (biceps, subscapular, suprailiac, thigh and calf) was measured to the nearest 0.5 mm using a caliper (SATA, Seville, Spain). The upper arm, waist, thigh, and calf circumferences, as well as their corresponding widths, were measured to 0.1 cm using a tape measure and sliding caliper (SATA, Seville, Spain). The subsequent average value of the three consecutive measures was obtained as a test result. BMI was commonly presented as a fraction of body weight (kg) and a square of the standing height (m^2^)

Carters and Heath’s [27] equation was used to distinguish somatotype features and correspondingly presented using a somatochart.

### 2.4. Maturity Status

To estimate maturity, Mirwald and colleagues [28] developed an equation for boys to estimate the years since their peak height velocity (PHV), which is an indicator of the adolescent growth spurt.

Maturity offset = −9.236 + 0.0002708 (leg length (cm) × sitting height (cm)) − 0.001663 (age (CA) × leg length (cm)) + 0.007216 (age (CA) × sitting height (cm)) + 0.02292 (weight (kg)/height (cm)).

Maturity offset represents the time before and after PHV and maturity was calculated by subtracting the age at PHV from chronological age. To overcome potential age effects [29], we used age-specific z-scores to classify players according to maturity status [30]. Using the age-specific z-scores of the predicted age at peak height velocity, players were categorized as “Early Maturers” (z < −1), “On time Maturers“ (−1 ≤ z ≤ 1), or “Late Maturers” (z > 1), in terms of their maturation status [31].

### 2.5. Statistical Analysis

The results were presented as means ± standard deviations (SD). The normality of data distribution was checked for all variables using the Kolmogorov-Smirnov test, while Levene’s test was used to check the homogeneity of variance. Pearson r correlation was used to estimate the relationship between age and body composition features. One-way between-groups analysis of variance (ANOVA) was carried out to investigate the differences in chronological age, body composition, and anthropometric measurements between three maturity groups. For a significant F ratio, the Tukey post hoc test was used to evaluate the differences among groups. The effect size was presented using partial eta square (η^2^) (η^2^ < 0.01 indicates a small effect; <0.06 medium effect; and <0.14 a large effect) [32]. Data analysis was conducted using the SPSS software package (IBM Corp. ver. 22.0), while the somatochart was created using Microsoft Excel (MS Excel 2021; Seattle, WA, USA). The level of statistical significance was set to the conventional 95% (*p* < 0.05).

## 3. Results

The classification of the sample according to maturity status showed that two-thirds (73.44%, n = 47) of football players were on time in maturing, 12.50% (n = 8) were early maturing and 14.06% (n = 9) were late maturing. The Kolmogorov–Smirnov test confirmed the normality of data distribution across all the variables, while the homogeneity of variance assumption was not disturbed. Moderate correlations were observed between age and body composition (Table S2). Table 1. shows the anthropometric and body composition characteristics and maturity status of the analyzed football players. Maturity, standing height, sitting height, leg length, FFM, and MM presented significant differences within the maturity status (*p* < 0.001), where all mean values were higher in early maturers when compared to their late maturing peers (Table 1). Skinfold thicknesses were significantly different (*p* < 0.05) for subscapular and suprailiac areas, where both mean values were lower in late maturers when compared with the other two groups (Table 2).

In general, girth increased with maturity status and showed statistically significant differences (*p* < 0.05) within the maturity group for all tested variables. Moreover, mean values for femur and humerus widths significantly differed within maturity status.

**Figure 1:**
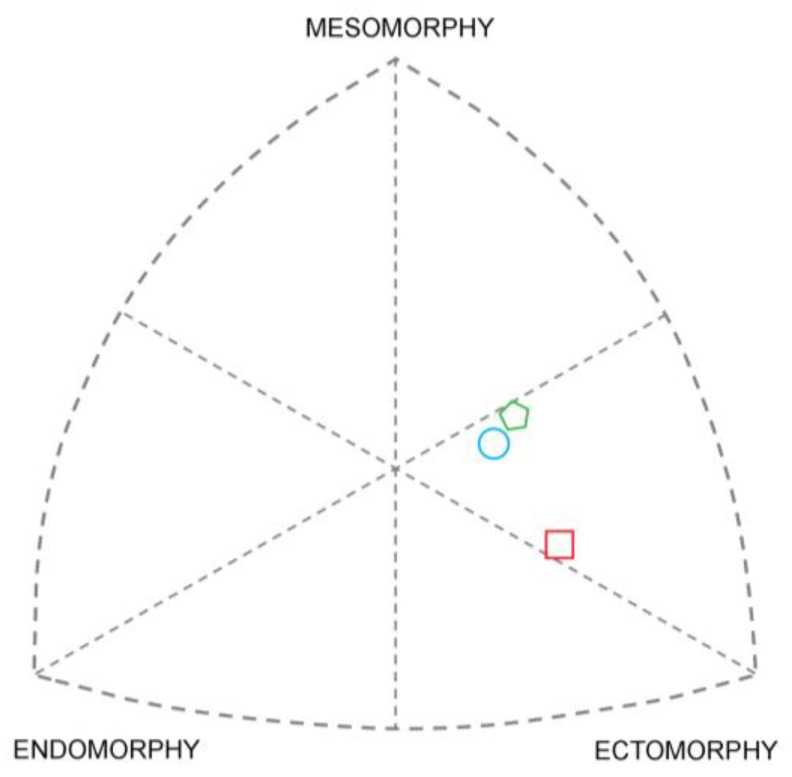
Somatochart of U15 players sub-grouped as early maturers (square), on-time maturers (circle), and late maturers (pentagon).

Regarding somatotype, there were no significant differences within maturity status, but it is evident that early maturers are balanced ectomorphs, while average maturers and late maturers are meso-ectomorphs (Figure 1). Mean differences with 95% CI across variables between different maturity levels are presented in Figure S1 and Table S1.

## 4. Discussion

The purpose of this study was to investigate the body dimensions, anthropometry, and somatotype-related differences in different body maturity levels among elite U15-level football players. This research of U15 level male football players found an advantage for early maturers with a more balanced ectomorphic somatotype, in addition to superior body composition and anthropometric characteristics. As a result, recognizing these ideal physical presentations in a player might play a part in getting the finest athletic performance.

Across the adolescent growth spurt, which occurs around this age, physical and physiological differences and maturity levels of players of the same chronological age fully emerge, which, therefore, impacts their sporting performance and their selection into the team at competitive levels. In the context of age at PHV, the effect of body maturity on physical performance testing for agility and speed has been observed in U14 and U15 level football players only [30]. The peak improvement in the crucial factor of aerobic capacity has also been noticed at around the age of PHV [33]. It has also been argued that chronological age influences the physical activity of players more than biological maturity [34].

Anthropometric characteristics and body composition is known to differ between adolescent subjects of the same chronological age based on their biological maturity [35]. The physical attributes, body composition, as well as somatotype of the players, are influenced by the body’s maturation status. Our results suggested the early maturers tended to have a dimensional superiority in standing and sitting height, and leg length, as described in Table 1. Lower body height in late maturers could hinder achieving the jumping height required for optimal ball heading, as has been pointed out by Bandyopadhyay, A [36]. Additionally, the lower body mass of late maturers prevents them from absorbing the greater momentum projected during body contact, unlike the early and on-time maturers.

The results showed significant differences for subscapular and suprailiac skinfolds across early, on-time, and late maturers. Moreover, early maturing football players demonstrated higher girth measurements, which may be attributed to the higher body dimensions. All these results are consistent with those of Rommers et al. [30], who further demonstrated higher sports performance in U15 early mature football players. An earlier review projected that the footballers should have an average body fat of 10% [37], which is in accordance with our study results, further differentiating into lesser BF% for early maturers (9.28%) than their late maturing (10.86%) counterparts. This corresponds with the amount of FFM, representing higher FFM for early maturers. The higher the player’s FFM or lean body mass, the greater the energy output and, therefore, the higher his cardiorespiratory fitness is, which reflects on his sports performance [38]. Similar conclusions have been reported, with larger physical dimensions having a positive influence on the performance of handball players [39]. In contrast, early mature males were found to have no significant height, weight, and other anthropometric advantages later into adulthood, as late maturers can subsequently catch up or even surpass their early maturing peers [40,41].

Football players are required to excel in speed as they need to run across the field while chasing the ball, thus it is recommended for them to be lighter and more efficient. This target can be optimally achieved through a combination of nutritional and training strategies plus hormonal factors and genetics. While football players are known to have the lowest body fat values, compared to other sports and non-training counterparts [35,42], it is evident from our results that the FFM and MM were significantly different within the three maturity levels, and that their mean values increased with the maturity status progression. Furthermore, the body fat percentage has been shown to be significantly related to the crucial parameters of speed and agility in young football players [43]. Therefore, it becomes obvious in adolescence that anthropometric characteristics play a significant role in specific sports performance. Studies have shown that an athlete’s body composition has a significant impact on his performance and that his body fat percentage is highly correlated with it [44].

Somatotype differences among the three groups were insignificant for the U15 football players, though the early maturers presented with balanced ectomorphic and average and late maturers with meso-ectomorphic somatotypes. It is probably attributed to the differential body fat percentage among the three maturity groups, with the early ectomorphic maturers presenting with the highest amount of FFM. Similar results were reported by Toselli et al. [35] who discovered that football players with early and on-time maturity presented with higher ectomorphic content. Few studies have reported conflicting results with a predominance of mesomorphy in young football players [45], and more frequent ectomorphy than in adult players [46]. Nikolaidis et al. [47] reported a shift in the somatotype paradigm across adolescence, with a decrease in endomorphy and ectomorphy, and thereby an increase in mesomorphy. In a previous study, 20–24-year-old football players were reported to be ectomorphic mesomorphs [38]. Our results are in line with this, as the U15 participants have primarily demonstrated the ectomorphic component, which may subsequently shift to mesomorphic with advancing age. The late-maturing participants in the present study obtained a meso-ectomorphic somatotype. With reference to the relationship between morphology and maturation reported by Malina et al. [48], the higher linearity is more characteristic of late than early maturers. Moreover, another important physical demand from a sport, such as football, is anaerobic performance, which may be positively affected by mesomorphic components associated with lean mass or the FFM [49]. This might be contributed to the fact that the fat-free mass index has a positive association with muscular strength and power, giving an overall advantage in sports performance. Since body composition and somatotype play a role in determining the player’s anaerobic capacities, such profiles can be a useful tool to develop an individualised training program, which could be further tailored to the specific tactical position on the field.

A previous study on elite male futsal players concluded that the injury rates were higher in players with predominantly endomorphic or mesomorphic features [50]. Therefore, having a clear understanding of the anthropometric and body composition profiles, coupled with maturation status in relation to the player’s somatotype can pave the path to facilitate the appropriate selection of football players by youth academies, lowering the injury rate and promoting long-term performance. The players with the most optimal physical and maturational presentation are the ones expected to perform the best, in a supporting psychosocial environment conducive to their overall development and successful game performance.

The present study reflected superior body dimensional and lower body fat profiles in young (U15) football players achieving early body maturity. This study data will serve as a frame of reference for the body composition and anthropometric scores in reference to the various somatotypes in adolescent football players (U15). However, this study did not consider these parameters with respect to their differential playing positions on the field. Further research is warranted to investigate whether different positional roles, requiring a specific skill-set, correspond to the players’ morphological presentation. Additionally, it would be interesting to determine whether and to what extent the somatotype or the body composition and maturation profile can serve a predicting role in injury occurrence because it could help youth academies and coaches to identify players who may be at a higher risk of injury and take appropriate measures to prevent injuries.

### 4.1. Strengths and Practical Implications of the Study

The study provides valuable information on the anthropometric and body composition profiles of young football players at different maturity levels, which can be useful for youth academies in selecting players and promoting long-term performance. The study also highlights the importance of body composition and maturation status in specific sports performance, which can inform training and nutritional strategies for young football players. Additionally, the study can serve as a reference standard for the body composition and anthropometric scores in reference to the various somatotypes in adolescent football players. Overall, the study contributes to the understanding of the physical characteristics of young football players and their implications for performance and development.

### 4.2. Limitations of the Study

Some potential limitations can be inferred, such as the small sample size, which may limit the generalizability of the findings. Additionally, the study only included football players from the first division in Bosnia and Herzegovina, which may limit the applicability of the results to other populations or levels of competition. Moreover, this study did not consider the physical performance of the players in relation to body dimensions and maturity. The exclusion criteria may also have limited the representativeness of the sample. Finally, the study design was observational and descriptive, which may limit the ability to establish causality or make predictions about future outcomes.

## 5. Conclusions

Football is a sport requiring both aerobic and anaerobic components in order to achieve best on parameters, such as speed, agility, power etc. This study, which investigated U15 level male football players, concluded that early maturers presenting with a more balanced ectomorphic somatotype have an edge, in addition to the superiority in body composition and anthropometric parameters. Therefore, the recognition of these optimal physical presentations in a player could serve a guiding role in achieving the best sporting performance.

## Figures and Tables

**Table 1:** Anthropometric and body composition characteristics by maturity status.

Variables	Early Maturers (n = 8)		On-Time Maturers (n = 47)		Late Maturers (n = 9)			Maturity (ANOVA)	
	Mean	SD	Mean	SD	Mean	SD	*η_p_* ^2^	*F*	*p*-Value
Age	14.45	0.36	14.25	0.20	14.08	0.11	/	15.006	0.000
Standing Height (cm)	179.10 ^€^	5.93	166.65 ^$^	5.86	149.57 ^£^	5.68	0.647	55.828	0.000
Sitting height (cm)	91.35 ^€^	3.02	85.00 ^$^	2.99	76.30 ^£^	2.89	0.645	55.800	0.000
Leg length (cm)	87.74 ^€^	2.90	81.64 ^$^	2.87	73.27 ^£^	2.78	0.646	55.722	0.000
Weight (kg)	61.75 ^€^	10.35	52.36 ^$^	6.39	38.81 ^£^	4.66	0.453	25.279	0.000
BMI (kg/m^2^)	19.17	2.09	18.60	2.32	17.41	1.40	0.048	1.533	0.224
FFM (kg)	55.73 ^€^	6.93	47.05 ^$^	5.33	34.56 ^£^	4.06	0.527	33.938	0.000
MM (kg)	52.90 ^€^	6.61	44.64 ^$^	5.08	32.75 ^£^	3.87	0.526	33.817	0.000
BF% (%)	9.28	3.80	10.11	2.61	10.86	3.14	0.021	0.650	0.525

SD = standard deviation; BMI = body mass index; FFM = fat-free mass; MM = muscle mass; BF% = percentage of body fat; *η_p_*^2^ = partial eta squared; ^€^ = sig. difference between early maturers and on-time maturers *p* < 0.05; ^$^ = sig. difference between on-time maturers and late maturers *p* < 0.05; ^£^ = sig. difference between early maturers and late maturers *p* < 0.05.

**Table 2:** Longitudinal and transversal dimensions by maturity status.

Variables	Early Maturers (n = 8)		On-Time Maturers (n = 47)		Late Maturers (n = 9)			Maturity (ANOVA)	
	Mean	SD	Mean	SD	Mean	SD	*η_p_* ^2^	*F*	*p*-Value
Skinfold thickness (mm)									
Biceps (mm)	6.75	1.98	8.00	2.32	6.33	1.41	0.087	2.90	0.063
Subscapular (mm)	6.87 ^€^	2.58	6.68	1.27	5.16 ^£^	1.17	0.124	4.60	0.018
Suprailiac (mm)	7.75	3.80	7.51 ^$^	2.28	5.00	1.80	0.121	4.19	0.020
Thigh (mm)	11.25	3.10	11.48	3.24	10.11	3.10	0.022	0.697	0.502
Calf (mm)	9.00	3.25	9.95	3.23	8.11	2.66	0.045	1.43	0.245
Girth (cm)									
Upper arm (cm)	23.77	2.50	23.14	2.49	21.16	1.29	0.095	3.18	0.048
Waist girth (cm)	73.05	6.12	70.29 ^$^	5.07	64.44 ^£^	3.98	0.184	6.86	0.002
Thigh girth (cm)	47.57	8.29	47.38 ^$^	3.05	43.27 ^£^	3.64	0.115	3.96	0.024
Calf girth (cm)	33.72	1.83	33.64 ^$^	2.21	30.16 ^£^	1.96	0.252	10.25	0.000
Breadth (cm)									
Humerus (cm)	7.56	0.79	7.36	0.78	6.73	0.41	0.095	3.17	0.047
Femur (cm)	9.14	2.64	9.08 ^$^	0.97	7.77 ^£^	1.15	0.252	3.95	0.023
Somatotype									
Endomorphy	2.19	1.02	2.55	0.75	1.99	0.59	0.074	2.43	0.096
Mesomorphy	2.40	1.09	3.66	1.78	3.59	1.53	0.044	1.41	0.250
Ectomorphy	4.71	0.93	4.09	0.80	3.81	0.84	0.081	2.68	0.077

SD = standard deviation; *η_p_*^2^ = partial eta squared; ^€^ = sig. difference between early maturers and on-time maturers *p* < 0.05; ^$^ = sig. difference between on-time maturers and late maturers *p* < 0.05; ^£^ = sig. difference between early maturers and late maturers *p* < 0.05.

## Data Availability

The data presented in this study are available upon reasonable request from the corresponding author.

## Supplementary Materials

The following supporting information can be downloaded at: https://www.mdpi.com/article/10.3390/children10040721/s1, Figure S1: Mean differences and 95% confidence intervals between early (n = 8), on-time (n = 47), and late (n = 9) maturers for height, body composition, skinfolds, girths, breadths, and somatotype characteristics in U15 elite football players; Table S1: Pearson r correlations between chronological age and body composition (BC) features in U15 football players (n = 64).
