# Supplementary material for: Maturity-Related Variations in Morphology, Body Composition, and Somatotype Features among Young Male Football Players
Source: Children (Basel). 2023 Apr 13;10(4):721. doi: 10.3390/children10040721 (PMC10136423; doi:10.3390/children10040721)
Supplement: Supplementary file 1 [file children-10-00721-s001.zip › children-2333062-supplementary.pdf]

children

MDPI

Supplementary Material

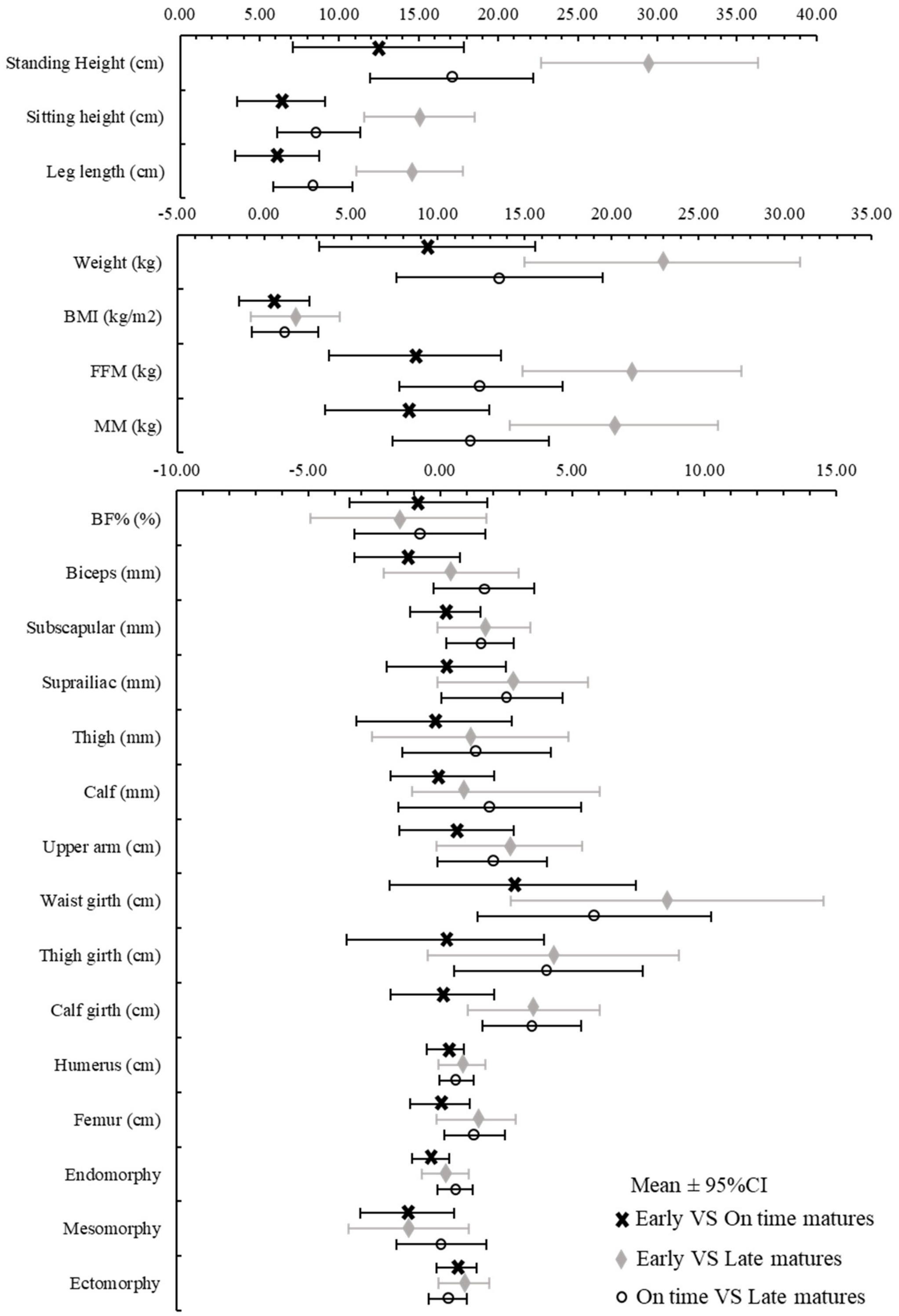

Figure S1. Mean differences and 95% confidence intervals between early (n = 8), on-time (n = 47), and late (n = 9) maturers for height, body composition, skinfolds, girths, breadths, and somatotype characteristics in U15 elite football players.

Table S1. Pearson r correlations between chronological age and body composition (BC) features in U15 football players (n = 64).

Age	Standing Height (cm)	Sitting Height (cm)	Leg Length (cm)	Weight (kg)	BMI (kg/m^2)	FFM (kg)	MM (kg)	BF% (%)
1	0.488 **	0.487 **	0.486 **	0.381 **	0.008	0.466 **	0.465 **	−0.415 **
